# Books Reviewed

**Published:** 1867-04

**Authors:** 


### Books Reviewed.

Contributions to the Pathology, Diagnosis, and Treatment of Angular Curvatures of the Spine. By Benjamin Lee, M. D. Philadelphia: J. B. Lippincott & Co., 1867.

Angular curvatures of the spine have recently received increased attention, and the former theories and measures of treatment have been subjected to careful revision. The author of this work speaks in the first place of gastralgia, as the initial symptom of caries of the spine, considering it at some length, and then passes to a consideration of certain errors in regard to the pathology and treatment of ulcerative inflammation of the spine, commonly called "Pott's Disease." This chapter has especial attractions, and cannot fail to interest all who bestow any attention upon the subject. In its careful perusal we find so much to approve, so many things which should be known to practitioners, that we really believe the book should be distributed universally to the profession, in the same manner that benevolent societies distribute tracts.

There is a great deal of injury done to the victims of this disease by ill-timed measures of treatment, and it is quite time some standard author furnished not only a reasonable guide, but a positive protest against prevalent practices. Our author has treated the subject of counter-irritation so charmingly, that we shall be forgiven, if we make a few selections from his work; we only regret that space will not permit to quote him entire; we are very glad to find such views entertained by so distinguished an author. He says: "During the past two years—in which I have been devoting myself more especially to the study of diseases of the spine and spinal cord--I have seen the integuments of the back seamed and scored and furrowed by the scars of the seton, the issue, the morax and the cautery, and by the remarkable regularity with which these appearances have been accompanied by serious, generally irremediable deformity. The question gradually forced itself upon me: Is this treatment, painful in its application and exhausting in its operation, sustained by the test of experience or founded on the basis of reason? The number of unfortunates whom I saw, who bore unmistakable traces of having fully tested the benefits of this mode, and in whom the disease was still unchecked, or checked only becuuse the deformity had reached its natural limit before the patient had succumbed to its deadly power, gave a sadly eloquent negative to the first clause of the question."

The causes of the disease are then discussed, whether it be dependant upon a tubercular diathesis, as is maintained by a certain class of practitioners, or whether it be the result of a traumatic cause. The author admits that *morbus caxarius* may arise in children whose constitutions have been vitiated by the sins of their ancestors, but his conviction is, that the disease is generally of the nature of a simple inflammation, often the result of external violence. In answer why counter-irritation is not allowable and advisable in the treatment of caries of the vertebræ, he remarks: "first, because a strict regard for the ethics of our art and an honest self-respect will not permit us to make use of any remedies, however harmless, upon mere theory, if experience has proved them to be inert. Still less may we suffer the beauty of a theory to lead us to the adoption of means which, after a fair trial, have failed to establish their efficacy, if their employment is necessarily attended by excruciating pain, nervous irritation and general exhaustion. The evidence of its utility, should be ample and overwhelming, which could lead us to initiate a mode of treatment which is in itself a disease, and which, were any of us suffering from it, would often be sufficient to incapacitate us from the discharge of our ordinary duties.

But, secondly, this treatment has not even the apology of a plausible and well-reasoned theory to bolster it up. Our standard writers, prominent among whom stands Pereira, are forced to acknowledge that all attempts to place counterirritation upon a rational foundation have entirely failed. While I can understand how an agent which stimulates the capillary circulation of the entire lower extremities may exert a derivative influence from distant parts, it does tax my powers of comprehension to appreciate how a small, circumscribed focus of suppuration, which possesses no controlling influence over the circulation can have any other effect, than simply to increase general nervous irritability.

Thirdly, and especially, this treatment is neither advisable nor admissable, because it consumes precious time, and prevents the employment of a means which is infinitely superior. The most that any man can claim for the result of his counter-irritation in any given case, is to fall back upon the miserable, conscience-salving subterfuge, 'If it had not been used, the patient might have been worse.'"

---

Injuries of the Spine, with an Analysis of nearly four hundred cases. By JOHN ASHHURST, JR., A. M., M. D., Fellow of the College of Physicians of Philadelphia, Surgeon to the Episcopal Hospital of Philadelphia, etc., etc. Philadelphia: J. B. Lippincott & Co., 1867.

The respective merits and demerits of treatment of injuries of the spine, by extension and counter-extension, resection and general treatment, are ably discussed by the author, who upon the analysis of 368 well authenticated cases arrives at the following general conclusions:

"1—Injuries of the spine are not nearly so fatal as is generally supposed, and they have been, not unfrequently, completely recovered from.

2—By watching carefully the symptoms and knowing the lesions which they indicate, the patient's progress towards health or death can be pretty accurately foreseen in most cases.

3—Whenever there is reason to believe that one or more vertebræ have been displaced, extension should be employed; temporary, if that be sufficient; if not, continuous.

4—In no case do resection or trephining offer a reasonable prospect of the patient's condition, but on the contrary there is reason to fear that they would increase the chances of a fatal termination.

5—Those cases of spinal injury which are not adapted for the employment of extension, should be treated in accordance with ordinary, rationai and physiological principles.

6—No new mode of treatment is entitled to adoption in a class of injuries so serious as this, unless it can be shown by clinical experience that it is at any rate *not less successful* than the modes commended to us alike by reason and long experience."

---

Medical Register of the District of Columbia, 1867, embracing notices of the Medical, Benevolent and Public Institutions of Washington. By J. M. TONER, M. D.

This little volume is designed by the author to give information of every organization, department or institution possessing any interest to the medical profession, or in any way related to medical affairs. It embodies much useful and interesting matter, not only to physicians of the District, but to the profession at large. Of especial interest will be found Senator Cowan's table, showing the physical characteristics of the U. S. Senators of the present session, which will be more fully noticed in our next issue. We trust that the author will receive from physicians that support which his enterprise merits.

